# Application of Calcium Hypochlorite for Sanitizing 3/16-Inch Tubing Used in Maple Sap Collection

**DOI:** 10.3390/microorganisms12101948

**Published:** 2024-09-26

**Authors:** Yangjin Jung, Olivia McHugh, Elijah Ayilaran

**Affiliations:** 1 Agricultural & Environmental Research Station, West Virginia State University, Institute, WV 25112, USA; olivia.mchugh@wvstateu.edu; 2 Department of Biology, West Virginia State University, Institute, WV 25112, USA; eayilaran@wvstateu.edu

**Keywords:** maple sap, tubing, calcium hypochlorite, biofilm, sanitation, SEM

## Abstract

Despite the widespread empirical adoption of calcium hypochlorite (Ca(ClO)_2_) for sanitizing 3/16-inch tubing after the maple sap collection season, there remains a dearth of scientific data on its best practice and effectiveness. To address this gap, we cultivated microbial mass in tubing through continuous maple sap flow at 7 °C for 5 weeks in the lab. The tubing was sanitized with 200, 400, or 600 ppm Ca(ClO)_2_ and retained Ca(ClO)_2_ for either 10 min, 1 h, 7 days, or 6 weeks. Half of the tubing segments underwent microbial analysis, while the other half were stored for 6 weeks post-flushing of the Ca(ClO)_2_ to determine microbial survival/growth. The level and presence of the microbial load were determined, and the inner tubing surfaces were visualized using scanning electron microscopy (SEM). The initial microbial load in the tubing was approximately 4–5 log CFU/cm^2^. A 10-min and 1 h contact time with 200 ppm Ca(ClO)_2_, and a 10-min exposure to 400 ppm Ca(ClO)_2_, achieved reductions of 2.4–2.8 log for *Pseudomonas* spp., 1.6–2.5 log for mold and yeast, and 2.3–3.3 log for psychrotrophic microorganisms. Microorganisms were recovered from the enrichment process after retaining 200 ppm Ca(ClO)_2_ for 6 weeks, indicating insufficient inactivation. Consequently, the data suggests the use of at least 400 ppm Ca(ClO)_2_ for 1 day. The SEM images supported the microbial count results, offering valuable insights for educating maple syrup producers on optimal tubing sanitation practices.

## 1. Introduction

Since the mid-1970s, the introduction of plastic tubing in maple sap collection has significantly enhanced operational efficiency over traditional methods involving buckets and sap bags, with the use of 5/16-inch diameter tubing connected to a vacuum pump being the industry standard. In the 2010s, the 3/16-inch tubing system was introduced, utilizing the creation of a natural vacuum given a sufficient elevation differential, thus eliminating the need for costly external devices like vacuum pumps, power sources, or releasers [1]. This innovation not only delivered economic and environmental benefits but also showcased improved sap flow rates in mountainous regions. However, challenges stemming from microbial accumulation within the narrow tubing after several seasons of use have arisen, resulting in diminished sap yields and lower syrup quality [2].

Perkins et al. [3], from the Proctor Maple Research Center and Cornell University, reported on research conducted between 2009 and 2018 regarding spout and tubing sanitation and its effects on sap yield and net profit improvement. Among different strategies, including spout replacement, use of leader check-valve, annual dropline change, sanitation with sodium hypochlorite (bleach), sanitation with hydrogen peroxide, sanitation with isopropyl alcohol (not allowed in the United States), and use of Zap-bac spouts, the use of check-valve type spout and sanitation with bleach showed promising results in increasing sap yield and net profit. In fact, the installation of new spouts, followed by the cleaning and sanitization of lines using a bleach solution after each season, led to a 75% increase in sap yield. While extensive research-based data exists regarding the sanitation of 5/16-inch tubing systems, knowledge regarding the sanitation practices specific to 3/16-inch tubing systems remains comparatively limited.

Since sodium hypochlorite degrades into salt, which can attract squirrels and lead to tubing damage during the off-season, maple sap producers have started opting for calcium hypochlorite (Ca(ClO)_2_) instead. Additionally, maple sap producers using 3/16-inch tubing have empirically adopted the “Krueger method”, which involves applying a 400 ppm concentration of Ca(ClO)_2_ [4]. The Krueger method developed by Arthur Krueger includes five key steps: (1) flushing mainlines first to avoid driving debris into lateral lines, (2) pumping or siphoning sanitizing solution into the lines, (3) sanitizing while pulling taps in the spring to remove residual sap, (4) leaving sanitizing solution in the lines throughout the summer, and (5) rinsing with water or sap before or as part of the first sap run of the coming season to flush out any remaining Ca(ClO)_2_. Moreover, spouts are sanitized by immersion in a sanitizing solution within a stericap, eliminating the need for annual replacements. This method has been shown to be effective based on field studies by Krueger, who observed visibly clean tubing, clearer sap yield, and no damage from squirrels [4,5].

Despite the widespread empirical adoption of the Krueger method by maple sap producers, there is a critical gap in scientific data validating its efficacy. Specifically: (1) What is the scientifically valid concentration to use? (2) What is the proper contact time? (3) Should the sanitation solution be retained or flushed after the appropriate contact time? To address these questions, this study artificially built up microbial mass in 3/16-inch tubing using continuous sap flow in a lab setting and assessed the efficacy of Ca(ClO)_2_ at different contact times and concentrations. Additionally, the sanitized tubing was stored for 6 weeks, with the Ca(ClO)_2_ either retained or flushed.

## 2. Materials and Methods

### 2.1. Building Up Microbial Mass in Tubing

**Figure 1:**
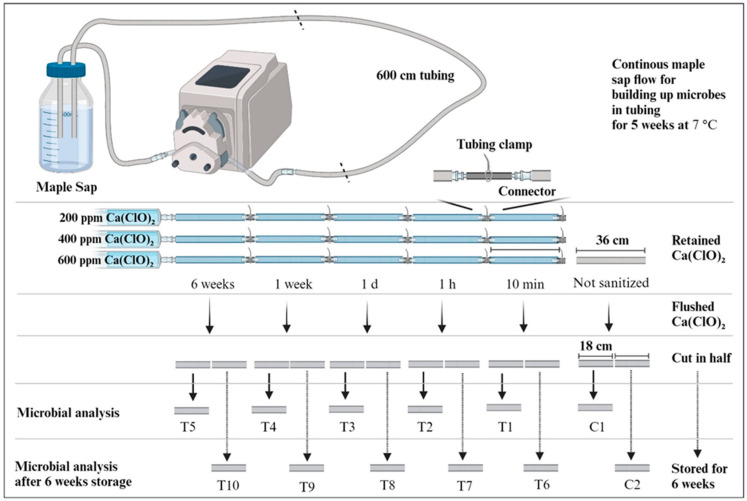
Experimental design for a lab-scale sanitation study. Figure created with BioRender.com.

Fresh maple sap was obtained from a research field site in Franklin, West Virginia, at the beginning of the maple sap collection season (31 January 2023) and stored at −20 °C. 500 mL of frozen sap was thawed in running tap water and connected to 600 cm of 3/16-inch semi-rigid sap collection tubing to build up microbial mass within the tubing (Roth Sugar Bush, Cadott, WI, USA). As shown in Figure 1, the sap was continuously flowed through the tubing at a rate of 2 mL/min using a peristaltic pump (BT100L, Golander Pump, GA, USA). The entire system, including the sap, tubing, and pump, was then placed in a 7 °C refrigerated incubator for a duration of 5 weeks. For comparison purposes, two 30 cm segments, aged two years and devoid of any sanitation, were acquired from a maple sap producer in Maine.

### 2.2. Lab-Scale Tubing Sanitation

As shown in Figure 1, following the 5-week period of building up microbial mass in the tubing, the 600 cm tubing was divided into 16 sections, each approximately 36 cm in length. Each set comprised five tubing pieces with connectors. These sets were connected to the peristaltic pump and filled with a solution of 200, 400, or 600 ppm of calcium hypochlorite (Ca(ClO)_2_; HTH^®^ Dry Chlorinator Granular, EPA Registration number: 1258–427). The level of free chlorine was measured using free chlorine test strips to ensure the efficacy of the Ca(ClO)_2_ (WaterWorks, Rock Hill, SC, USA). After filling with the sanitizing solution, the tubing clamp between tubing pieces was securely closed. At specified time intervals (10 min, 1 h, 1 d, 1 week, and 6 weeks), the connector part was detached from the system to flush out the Ca(ClO)_2_. Subsequently, the flushed tubing was cut in half; one portion (C1, T1–T5) was processed to recover microorganisms within the tubing, while the other portion (C2, T6–T10) was stored at room temperature for 6 weeks. After 6 weeks of storage, the tubing was processed to recover microorganisms.

### 2.3. Recovery of Microorganisms in Tubing

**Figure 2:**
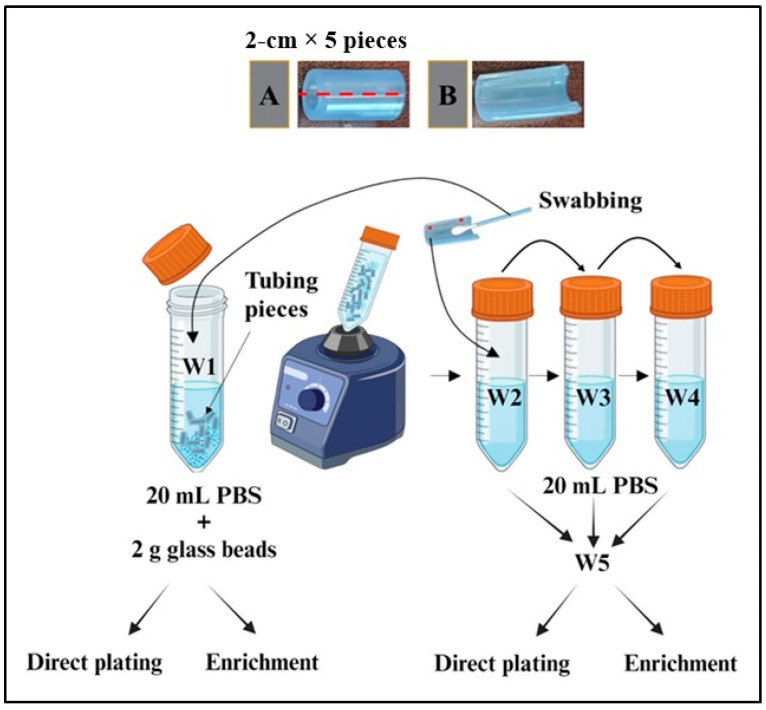
Recovery of microorganisms from the tubing. (**A**) A 10-cm tubing was cut into five 2-cm pieces. (**B**) The five tubing pieces were then halved. Figure created with BioRender.com.

To eliminate any microorganisms on the outer surface, the outside surface of tubing was sanitized; the tubing was wrapped with a paper towel moistened with 10% bleach for 2 min. Subsequently, the tubing was positioned vertically, and 70% alcohol was sprayed on the outside to remove the bleach and sanitize the tubing’s exterior, ensuring that no alcohol entered the tubing’s interior. Following this, each tubing was divided into segments measuring 10 cm and 1 cm, with the end parts excluded. The 10 cm segments were utilized for enumerating microorganisms, while the 1 cm tubing was processed for visualization of the interior using scanning electron microscopy (SEM). A glass beads–vortexing–swabbing method, as described by Kim et al. [6], Lagacé et al. [7], and Mandakhalikar et al. [8], was employed with modifications to recover microorganisms from the interior surface of the tubing (Figure 2).

Initially, a 10-cm sanitized tube section was cut into five 2-cm pieces (Figure 2A). The five tubing pieces were then halved (Figure 2B), resulting in 10 pieces. These segments were placed in a 50 mL tube (Figure 2, W1) containing 2 g of 3-mm glass beads (Supelco, Burlington, MA, USA) and 20 mL of phosphate-buffered saline (PBS; EMD Millipore, Burlington, MA, USA). After vortexing at maximum speed using an Analog vortex mixer (VWR, Radnor, PA, USA) for 1 min, the interior of all the tubes was swabbed back and forth 10 times with a cotton swab using plastic handled cotton swabs (Fisherbrand™, Pittsburgh, PA, USA). The swabs were then immersed in the W1. Subsequently, the tubing pieces were sequentially transferred to three separate 50 mL tubes (W2-W4) containing 20 mL of PBS for the rinsing process.

The PBS from W2-W4 was combined (W5), and an aliquot of W1 and W5 was plated in duplicate on dichloran rose bengal chloramphenicol (DRBC; BD, Sparks, MD, USA), *Pseudomonas* agar with CFC selective supplement (Cetrimide, fucidin, and cephaloridine) (P-CFC) (Sigma-Aldrich, St. Louis, MO, USA), and tryptic soy agar (TSA; BD, Sparks, MD, USA) to enumerate mold and yeast, *Pseudomonas* spp., and psychrotrophic bacteria, respectively. The DRBC, P-CFC, and TSA plates were then incubated at room temperature, 30 °C, and 7 °C for 5 d, 2 d, and 10 d, respectively. Furthermore, 10 mL of each W1 and W5 samples, along with rinsed tubing pieces, were combined and incubated with 20 mL each of double-strength tryptic soy broth (TSB; BD, Sparks, MD, USA) at 35 °C for 48 h. This enrichment aimed to recover any injured cells or confirm the presence/absence of microorganisms that may be below the detection limit (BD1; 0.83 log CFU/cm^2^).

### 2.4. Scanning Electron Microscopy (SEM)

The 1-cm tubing segments were fixed, rinsed, and dehydrated following the protocol of Lagacé et al. (2006) [7] with slight modifications. Initially, the tubing was immersed in 100 mM sodium cacodylate buffer (pH 7.4) containing 2.5% glutaraldehyde (Electron Microscopy Sciences, Hatfield, PA) for 2 h. Subsequently, the tubing underwent five rinses with 100 mM sodium cacodylate buffer, each for 15 min. Following rinsing, the samples were serially dehydrated with ethanol (30%, 50%, 70%, 90%, and three times with 100% ethanol in 100 mM sodium cacodylate buffer), with each dehydration step taking 15 min. The dehydrated samples were then dried for 15 min using hexamethyldisilazane (HMDS) as the drying agent, and then kept under vacuum in a desiccator until the moment they were sputter coated (Denton Desk V, Denton Vacuum, Moorestown, NJ, USA) with an ultrathin conductive film of gold–palladium before visualization using a JEOL JSM-7600F scanning electron microscope (Jeol, Akishima, Tokyo, Japan).

### 2.5. Data Analysis

The lab-scale microbial mass buildup and sanitation study was conducted independently twice. Microbial counts were expressed as logarithms of the number of colony-forming units (CFU) per cm^2^ of tubing, with microbial data reported as mean ± standard deviation. In cases where no colonies were detected through direct plating, microbial presence was assessed by observing turbidity compared to a sterilized growth medium blank (TSB). Since visible turbidity typically indicates microbial growth during the enrichment process, but the initial level cannot be quantified, turbid TSB was recorded as “<0.83 log CFU/g (detection limit)”, whereas clear TSB, indicating no growth, was recorded as “not recovered”. In other words, “not recovered” indicates that no culturable cells were observed through the enrichment process. The SEM images were captured at various magnifications (×50 to ×4000), and images that best depicted the overall tubing surface were selected for representation.

## 3. Results

### 3.1. Microbial Mass in Tubing

**Figure 3:**
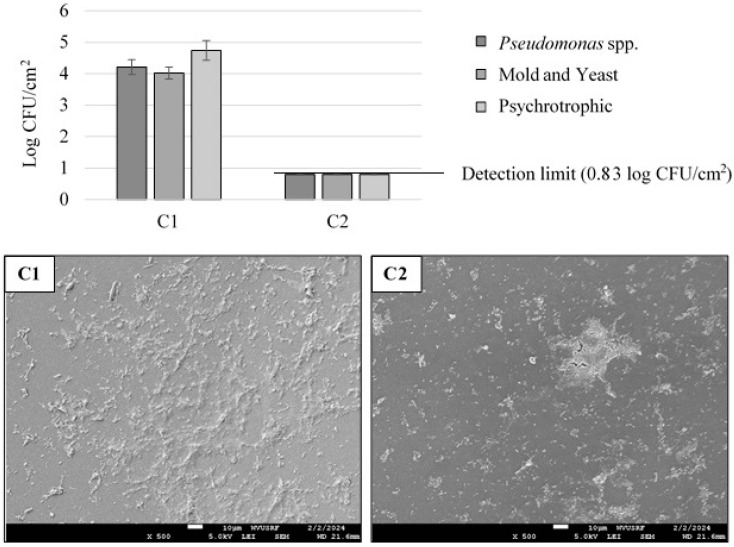
Microbial counts and visualized microbial mass accumulation inside 3/16-inch tubing in a lab-scale setting. **C1**: microbial accumulation with continuous maple sap flow at 7 °C for 5 weeks. **C2**: C1 tubing stored at room temperature for 6 weeks.

The initial microbial counts from the frozen sap were 3.5 ± 0.3, 3.0 ± 0.5, and 4.3 ± 0.2 log CFU/mL for *Pseudomonas* spp. mold and yeast, and psychrotrophic microorganisms, respectively. Figure 3 shows the initial microbial count (C1) and the C1 tubing that was stored at room temperature for 6 weeks (C2). Continuous sap flow resulted in microbial accumulation, with the following log CFU/cm^2^ values: 4.2 ± 0.2 for *Pseudomonas* spp., 4.0 ± 0.2 for mold and yeast, and 4.7 ± 0.3 for psychrotrophic microorganisms, as observed in C1 of the SEM image. After storing the C1 tubing at room temperature for six weeks, no viable microbial counts were detected using the direct plating method (C2). However, following an enrichment process at 37 °C for 48 h, microbial growth was observed, indicated by a change in turbidity. Additionally, some microorganisms were visualized using scanning electron microscopy (SEM). Consequently, as the worst-case scenario, the 0.8 CFU/cm^2^ values (C2) obtained following enrichment were presented in Figure 3. Due to the extended storage of the tubing, the microbial cells seemed to have experienced limited nutrients and dry conditions, causing their loss in recovery through a traditional direct plating method. Nevertheless, examination of the SEM images indicated that microorganisms were still present on the interior of the tubing.

### 3.2. The Efficacy of Calcium Hypochlorite

**Figure 4:**
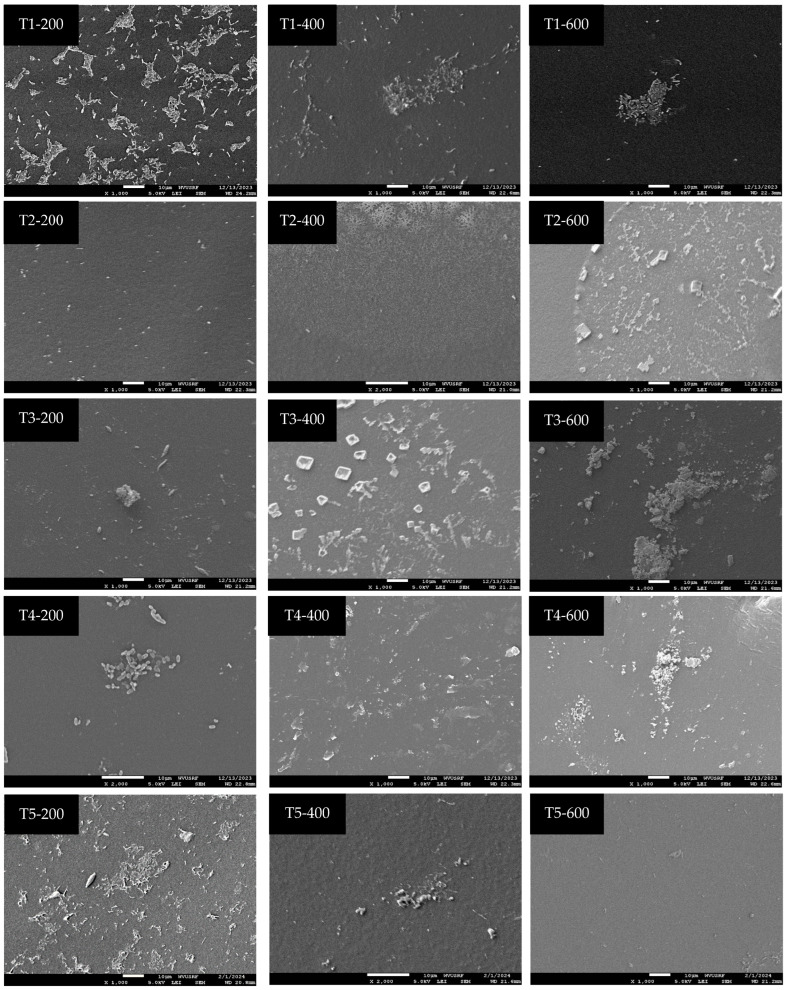
Representative scanning electron microscopy images showing the efficacy of sanitation with different contact time and concentration of Ca(ClO)_2_.

**Figure 5:**
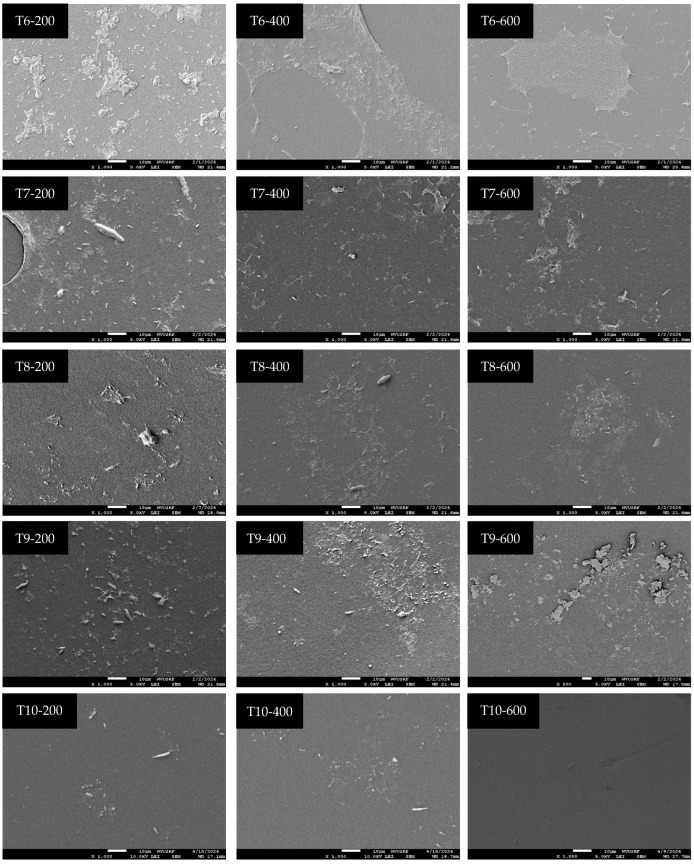
Representative scanning electron microscopy images showing the effectiveness of sanitation at varying contact times and concentrations of Ca(ClO)_2_, followed by a six-week storage period post-sanitation.

**Figure 6:**
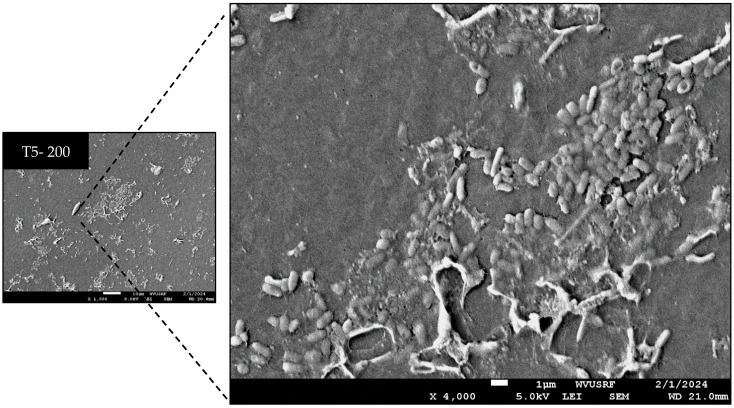
Enlarged image of T5-200. Visualized image of tubing treated with 200 ppm calcium hypochlorite Ca(ClO)_2_ and stored for 6 weeks.

Table 1 presents the efficacy of calcium hypochlorite (Ca(ClO)_2_) in sanitizing tubing at various contact times and concentrations, and Figure 4 and Figure 5 provide visualizations of the tubing samples’ interiors, corresponding to the data in Table 1. Sanitizing with 200 ppm Ca(ClO)_2_ for 10 min (T1-200) and 1 h (T2-200) resulted in reductions of 2.4–2.9 log, 1.6–2.2 log, and 2.3–3.3 log for *Pseudomonas* spp. mold and yeast, and psychrotrophic microorganisms, respectively, compared to the initial microbial load. Sanitizing with 400 ppm Ca(ClO)_2_ for 10 min (T1-400) achieved reductions of 2.6 log, 2.6 log, and 2.8 log for *Pseudomonas* spp. mold and yeast, and psychrotrophic microorganisms, respectively. Sanitizing with 600 ppm Ca(ClO)_2_ for 10 min (T1-600) effectively inactivated microorganisms within the tubing, as evidenced by the lack of recovery in the enrichment process. However, observation of the SEM image (T1-600 in Figure 4) revealed some microorganisms are present. This suggests that, while microbial cells were present, they were likely injured or in a non-culturable state and thus failed to be resuscitated by culturing with media. Interestingly, sanitizing with 200 ppm Ca(ClO)_2_ for 6 weeks (T5-200) did not completely eliminate microorganisms, as indicated by their growth after the enrichment process. Moreover, the microbial data (Table 1) and SEM images of T2-200 (Figure 4) and T7-200 (Figure 5) show re-colonization after 6 weeks of tubing storage. The SEM images were selected to provide an overview of the sanitizing efficacy. However, an enlarged image of T5-200 was presented in Figure 6 due to an unexpected result: microbial growth was observed after sanitizing with 200 ppm for 6 weeks.

The SEM images also revealed remaining crystals/clumps of Ca(ClO)_2_ residue in tubing samples T2-400, T2-600, T3-400, T3-600, T4-400, and T4-600. However, tubing treated with 400 ppm and 600 ppm Ca(ClO)_2_ solutions for 6 weeks did not exhibit residual Ca(ClO)_2_ (T5-400 and T5-600 in Figure 4). After 6 weeks of storage following the flushing of Ca(ClO)_2_ solution from the tubing, microbial presence was observed in samples T6-200, T7-200, T8-200, T9-200, T6-400, and T7-400, and residue of Ca(ClO)_2_ was observed in samples T9-400, T6-600, T7-600, T8-600, and T9-600.

**Figure 7:**
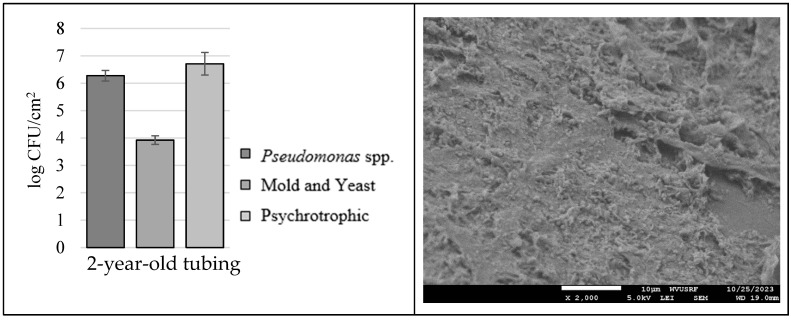
Microbial load and visualized microbial mass in 2-year-old 3/16 tubing from maple sap production field.

Figure 7 displays the microbial load in 2-year-old 3/16-inch tubing that did not receive any sanitation collected from a maple sap production field: 6.3 ± 0.2 log CFU/cm^2^ for *Pseudomonas* spp., 3.9 ± 0.2 log CFU/cm^2^ for mold and yeast, and 6.7 ± 0.4 log CFU/cm^2^ for psychrotrophic counts. As expected, the microbial load in tubing that has been in the field for two years (and thus used for two seasons) was higher than what is in tubing artificially grown in a lab setting for 5 weeks.

## 4. Discussion

It is well known that microbial contamination in maple sap can affect the sap yield and physicochemical and sensory properties of maple syrup, and thus its quality [9]. Cleaning and sanitizing tubing can be accomplished using tubing washing machines that combine air and high-pressure water or by dipping them in solutions containing sodium/calcium hypochlorite or food-grade hydrogen peroxide or isopropyl alcohol (not allowed in the United States). According to the North American Maple Syrup Producers manual [10], using bleach to sanitize tubing systems can yield increased sap results (around 76%) with proper contact time. The manual suggests sodium hypochlorite (house bleach) should be at 200 ppm, with contact times from 5 min to several hours. Perkins et al. [3] conducted a multi-year field study at University of Vermont maple Research center to compare the 3/16-inch and 5/16-inch maple tubing on sanitation and/or sap yields. They found the sanitation practices differ significantly between 5/16-inch and 3/16-inch tubing systems in maple sap production, leading to varying impacts on sap yields over time. They emphasized that strategies to mitigate microbial effects and achieve adequate sanitation vary between 3/16-inch and 5/16-inch tubing systems. While extensive research supports sanitation in 5/16-inch systems, there is limited information available for 3/16-inch systems. Thus, this study evaluated the efficacy of Ca(ClO)_2_ with different concentration and contact time in 3/16-inch tubing. Lagacé et al. [7] scientifically identified the composition of maple sap microflora and biofilm in the collection tubing system during the sap collection season. Their study demonstrated that bacterial populations in sap and on tubing reached 5 log CFU/mL or cm^2^ of psychrotrophic and *Pseudomonas* counts by 75% of the way through the season. The contamination was primarily from aerobic psychrotrophic bacteria, with a significant proportion of *Pseudomonas* spp. Consequently, the current study evaluated the levels of psychrotrophic bacteria and *Pseudomonas* spp. in maple sap collection tubing after sanitation with Ca(ClO)_2_. Additionally, a wide range of molds and yeasts have been identified in maple sap, with their overgrowth causing quality defects [11]. Therefore, the levels of mold and yeast inside of the tubing were also determined in this study.

Biofilm is one of the survival techniques for microorganisms causing unfavorable conditions in the food and agricultural industries. Once biofilms form, microorganisms become resistant to harsh environments, and it is hard to remove them [12]. Therefore, proper sanitation practices are required to prevent the formation of biofilms. This current study revealed that sanitizing tubing with 200 ppm Ca(ClO)_2_ for 10 min (T1-200) and 1 h (T2-200) resulted in 1.6–2.9 log reductions compared to the initial microbial load, however, sanitizing with 200 ppm Ca(ClO)_2_ for 6 weeks (T5-200) did not completely eliminate microorganisms which led re-growth after the 6-week period. As depicted in Figure 4, increased microbial presence was noted inside the tubing compared to T4-200. Specifically, in Figure 6, while some rod-shaped bacteria remained, certain cells appeared damaged, suggesting that maintaining 200 ppm Ca(ClO)_2_ for 6 weeks resulted in the inactivation of some microorganisms while injured cells were regrown during the extended period of time. The effectiveness of Ca(ClO)_2_ likely diminished over time, possibly due to its initial action and prolonged contact with organic materials. However, the Ca(ClO)_2_ solution might have mitigated water limitation or desiccation, allowing some injured cells to recover. This suggests that using 200 ppm is insufficient for sanitizing 3/16-inch tubing. In the case of sanitizing with 400 ppm Ca(ClO)_2_ for 10 min (T1-400), it achieved reductions of 2.7–2.8 log for microorganisms but was not able to completely inactivate the microorganisms shown in the SEM image. Indeed, under environmental stresses like extreme temperatures, pH, UV irradiation, desiccation, chemicals, water limitations, fluctuating oxygen levels, and nutrient deprivation, microorganisms may enter the viable but non-culturable (VBNC) state. Once these stressors are removed, these microorganisms can transition back to a culturable state [13,14]. These injured microorganisms could potentially regrow if sufficient nutrients and suitable environmental conditions are provided such as when a new sap collection season begins and fresh sap flows.

Ca(ClO)_2_ reacts with water to produce hypochlorous acid (HOCl), which is a strong oxidizer and disinfectant that inactivates microorganisms [15]. After this initial reaction, efficacy may be affected by several factors, including sunlight, organic compounds, pH, and temperature [16]. Thus, efficacy can vary based on these factors and microbial levels on the inner surfaces of tubing. Additionally, the length of time the tubing retains the sanitizing solution and tubing length can influence the efficacy of the sanitizer. The EPA indicates a maximum usage of 600 ppm Ca(ClO)_2_ on porous food contact surfaces, as indicated in its registration document for Ca(ClO)_2_.

Ca(ClO)_2_ is generally used to control biofilms in drinking water systems [17]. Zhang et al. [18] studied the effects of chlorine on preventing drinking water biofilms and discovered that the type of pipe material influenced the composition of the biofilm community. Additionally, Buse et al. [19] reported that the opportunistic pathogen *Legionella pneumophila* was still present after 30 min of chlorine disinfection. While Shen et al. [20] observed that long-term exposure to disinfectants could actually promote biofilm formation, Mathieu et al. [21] demonstrated that shock chlorination with 10 mg/L chlorine for 1 h effectively reduced the cohesiveness of 2-month-old biofilms, enhancing control by decreasing contact points in the extracellular polymeric substance (EPS) network.

Based on our findings, maple producers using 3/16-inch tubing systems are advised to consider this sanitation option: sanitizing with at least 400 ppm calcium hypochlorite solutions with sufficient contact time of 1 h or more followed by thorough flushing during the off-season. One of limitations of this study was that the initial microbial mass in the tubing was lower than what is typically found in real-world settings. In this study, microbial mass was built up at 7 °C for 6 weeks. However, the tubing used in this study was obtained from the field and was two years old, potentially exposed to higher temperatures during the summer. Elevated temperatures and the continuous flow of freshly produced sap in field conditions could result in increased microbial growth due to greater nutrient availability and more favorable conditions. Therefore, the effectiveness of the sanitizing method evaluated in the lab setting may be overestimated when compared to the initial contamination levels observed in the field. Another limitation of this study was the lack of analysis of residual Ca(ClO)_2_ in relation to the SEM images and microbial data. Since residual Ca(ClO)_2_ was detected in the SEM images, providing data on its concentration remaining in the sap and on tubing surface could offer a clearer explanation related to its efficacy. Consequently, based on these research findings, a multi-year field study is warranted. The study should involve recording residual Ca(ClO)_2_ levels over time, collecting tubing and sap samples from the field across different seasons, analyzing microbial levels and diversity in both tubing and sap samples to assess quality, and evaluating sap yield to determine the effectiveness of this sanitizing method.

## 5. Conclusions

Some sap producers may not frequently replace their tubing due to associated costs without sanitation. However, the lack of robust sanitation practices forces producers to frequently replace tubing, leading to increased costs for labor and materials and negatively impacting the environment due to plastic waste. Our objective was to provide scientific insights into the effectiveness of using Ca(ClO)_2_, thereby offering valuable information to both the scientific community and the maple syrup industry. This research has filled existing knowledge gaps and facilitated evidence-based decisions in maple sap collection and sanitation practices. The Kruger method, using 400 ppm Ca(ClO)_2_ for 10 min, achieved reductions of 2.6 log for *Pseudomonas* spp., 2.6 log for mold and yeast, and 2.8 log for psychrotrophic microorganisms. However, SEM images correspond with the direct plating results, suggesting potential recolonization of the tubing surface by surviving microbes. Given the potential for microorganism regrowth with a 200 ppm Ca(ClO)_2_ solution if retained for an extended period (6 weeks), particularly with short contact times (10 min to 1 h), it is recommended to flush the solution after use. However, with a 400 ppm Ca(ClO)_2_ solution and a contact time of more than 1 day, or with a 600 ppm Ca(ClO)_2_ solution, either flushing or retaining the solution for up to 6 weeks does not significantly impact microbial levels inside the tubing. The enumeration data and visual images can be used to educate maple syrup producers on optimal tubing sanitation practices.

## Figures and Tables

**Table 1:** The recovery of microorganisms after sanitation with calcium hypochlorite (Ca(ClO)_2_) in a lab-scale setting.

	Retain Ca(ClO)_2_	Storage after Flushing Ca(ClO)_2_	Log CFU/cm^2^		
			Ca(ClO)_2_		
			200 ppm	400 ppm	600 ppm
T1	10 min	-	* PSD: 1.8 ± 0.3 MY: 2.4 ± 0.2 PSY: 2.4 ± 0.3	PSD: 1.4 ± 0.1 MY: 1.9 ± 0.3 PSY: 1.4 ± 0.3	Not Recovered
T2	1 h	-	PSD: 1.6 ± 0.1 MY: 1.5 ± 0.2 PSY: 1.9 ± 0.3	<0.83 /Not Recovered	Not Recovered
T3	1 d	-	<0.83 ^§^	Not Recovered *	Not Recovered
T4	1 Week	-	<0.83	Not Recovered	Not Recovered
T5	6 Weeks	-	<0.83	Not Recovered	Not Recovered
T6	10 min	6 Weeks	<0.83	<0.83	Not Recovered
T7	1 h	6 Weeks	<0.83	<0.83	Not Recovered
T8	1 d	6 Weeks	<0.83	Not Recovered	Not Recovered
T9	1 Week	6 Weeks	Not Recovered	Not Recovered	Not Recovered
T10	6 Weeks	6 Weeks	Not Recovered	Not Recovered	Not Recovered

Values represent the mean ± standard deviation (*n* = 2). * PSD: *Pseudomonas* spp. counts; MY: Mold and yeast counts; PSY: psychrotrophic counts. ^§^ <0.83: Below the detection limit; colony was not detected by using direct plating method, but microbial growth was observed following enrichment in TSB for 48 h at 37 °C. * Not recovered: No microbial growth was observed following the enrichment process.

## Data Availability

The original contributions presented in the study are included in the article, further inquiries can be directed to the corresponding author.
